# Integrating Biological Maturity into Fitness Assessment and Physical Activity Interventions in Children and Adolescents: A Narrative Review

**DOI:** 10.3390/sports14050196

**Published:** 2026-05-09

**Authors:** Souhail Bchini, Ismail Dergaa, Wissem Dhahbi, Halil İbrahim Ceylan, Valentina Stefanica, Taoufik Selmi, Dhouha Moussaoui, Nadhir Hammami

**Affiliations:** 1 Research Unit “Sport Sciences, Health and Movement” (UR22JS01), High Institute of Sport and Physical Education of Kef, University of Jendouba, Kef 7100, Tunisia; souhail.bchini@gmail.com (S.B.); phd.dergaa@gmail.com (I.D.); wissem.dhahbi@gmail.com (W.D.); nadhir.hammami@issepkef.u-jendouba.tn (N.H.); 2 Higher Institute of Sport and Physical Education of Ksar Said, University of Manouba, Manouba 2010, Tunisia; taoufikselmi72@gmail.com (T.S.); douhamoussaoui@yahoo.fr (D.M.); 3 Physical Activity, Sport and Health Research Unit, UR18JS01, National Observatory of Sport, Tunis 1003, Tunisia; 4 Physical Education of Sports Teaching Department, Faculty of Sports Sciences, Ataturk University, Erzurum 25240, Türkiye; 5 Department of Physical Education and Sport, Faculty of Sciences, Physical Education and Informatics, National University of Science and Technology Politehnica Bucharest, 060042 Pitești, Romania

**Keywords:** biological maturation, physical fitness, youth sport, peak height velocity, bio-banding, performance assessment, physical activity interventions, sex differences

## Abstract

Background: Childhood and adolescence represent critical developmental periods characterized by rapid somatic growth, endocrine changes, and the progressive attainment of biological maturity. These maturational processes substantially influence the development of physical fitness, yet are often overlooked when evaluating performance in youth populations. This structured narrative review aims to synthesize current knowledge regarding the relationships between somatic growth, biological maturity, and physical fitness in children and adolescents. Methods: A structured narrative review was conducted by systematically searching PubMed, Scopus, and Web of Science databases for peer-reviewed articles published up to February 2026. Keywords included ‘biological maturation’, ‘physical fitness’, ‘youth’, ‘adolescence’, ‘peak height velocity’, and ‘bio-banding’. Studies were eligible if they examined relationships between biological maturity indicators and physical fitness outcomes in children and adolescents aged 8–19 years. No publication date restrictions were applied, although priority was given to articles from the past 15 years. Results: Evidence consistently indicates that biological maturity substantially influences muscular strength, power, and speed in males; findings among females and for cardiorespiratory fitness are more nuanced and context-dependent. Early-maturing boys typically exhibit superior strength and power performances, whereas findings among girls are more variable. Earlier maturation in girls is frequently associated with increased adiposity, which may attenuate performance in weight-bearing activities. When maturity status is ignored, physical fitness evaluations may misrepresent the capabilities of late-maturing youth and potentially discourage long-term participation in physical activity. Conclusions: Integrating biological maturity into youth fitness evaluation frameworks is essential for accurately interpreting performance data and for providing developmentally appropriate interventions. Three implementation strategies are recommended: (i) adoption of maturity offset or percentage of predicted adult stature as standard covariates; (ii) development of maturity-stratified normative standards; and (iii) implementation of bio-banding in youth sport development.

## 1. Introduction

Understanding the development of physical fitness during youth requires careful consideration of the biological processes that occur throughout growth and maturation. Late childhood and adolescence are among the most dynamic phases of human growth and development, typically spanning ages 8 to 19 [1]. This age range was selected because it encompasses the full pre-pubertal, pubertal, and post-pubertal transition in both sexes [1,2], and represents the window during which maturation-related variability in physical performance is most pronounced and practically consequential [3]. During this interval, individuals experience a cascade of biological transformations triggered by pubertal endocrine activity. These processes result in rapid increases in stature, alterations in body composition, and progressive maturation of the musculoskeletal and cardiovascular systems. Importantly, the timing and tempo of these developmental changes vary widely between individuals [4]. As a result, adolescents of the same chronological age may differ considerably in their biological maturity and, consequently, in their physical capabilities [3].

Physical fitness refers to a collection of attributes that enable individuals to perform physical activity effectively [5]. For this review, fitness is conceptualized within a dual framework: (1) health-related fitness, encompassing cardiorespiratory endurance, muscular strength and endurance, flexibility, and body composition—components that reflect physiological capacity and long-term health status; and (2) performance-related fitness, including speed, agility, and power, which are particularly relevant to youth sport and talent identification contexts. This inclusive framework is justified by the dual applied scope of the review, which addresses both public health programming and sport development settings. Monitoring these attributes during youth is important for assessing health status, guiding physical education programs, informing sport participation, and predicting long-term health outcomes [6,7,8,9]. However, interpreting fitness data in this age group presents challenges because large inter-individual differences in biological maturity can strongly influence test performance.

Biological maturation describes the process through which an individual progresses toward a mature biological state [2]. In pediatric populations, maturity status is commonly evaluated using indicators such as skeletal age, secondary sexual characteristics (Tanner stages), or prediction models estimating maturity offset or the proportion of adult stature already achieved. Each method provides useful information but also carries methodological limitations that must be considered in both research and applied contexts [10,11].

Despite growing recognition of maturation-related variability in youth fitness, the field lacks a unified framework that simultaneously addresses assessment methodology, sex-specific patterns, and intervention design. Previous systematic reviews have addressed specific sub-questions: Mills et al. [12] evaluated the accuracy of PHV prediction methods but did not address fitness outcomes; Albaladejo-Saura et al. [13] examined maturation–fitness relationships without integrating intervention design; and Parry et al. [14] focused on injury associations with growth, excluding broader fitness domains and public health programming. None of these reviews synthesized all three domains—assessment methodology, sex-specific fitness patterns, and applied intervention design—within a single conceptual framework. This narrative review addresses this gap by integrating contemporary evidence across these interconnected domains and by proposing a conceptual model to guide future research and practice.

The specific aims of this review are to (i) synthesize evidence on the relationships between biological maturity and each of the five core physical fitness components in children and adolescents aged 8–19 years; (ii) examine sex-specific patterns and methodological considerations in maturity assessment; and (iii) propose evidence-based recommendations for integrating maturity into physical education, talent identification, and public health programming. The review is deliberately cross-contextual in scope, encompassing three overlapping population settings: (a) school-based physical education and population-level public health programs; (b) youth sport development and talent identification pathways; and (c) general pediatric exercise science research, including both athletic and non-athletic samples.

**Figure 1:**
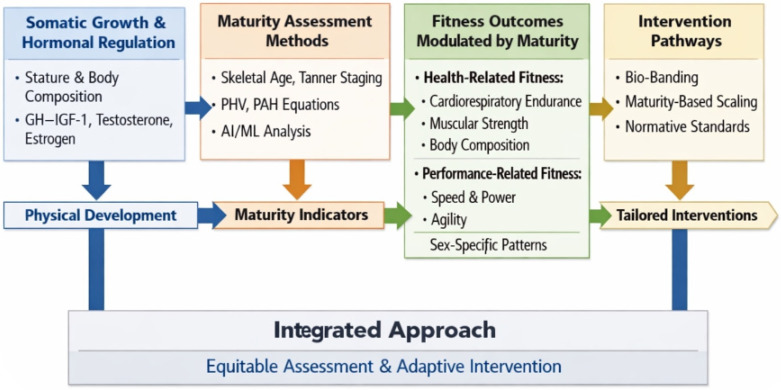
Conceptual Model for Integrating Biological Maturity into Fitness Assessment and Physical Interventions in Children and Adolescents.

To address this fundamental shortcoming and guide future research, we drew on the broader literature on integrating biological maturity into fitness assessment and physical interventions in children and adolescents to develop a conceptual mechanistic model (Figure 1). Underpinning this review, the model integrates biological maturity into intervention and measurement frameworks through four interconnected domains: (1) somatic growth and hormonal regulation, which shape body size, composition, and functional capacity; (2) maturity assessment methods, such as skeletal age, Tanner staging, and maturity offset equations, which categorize early, average, and late matures; (3) fitness outcomes, including cardiorespiratory endurance, muscular strength, flexibility, speed, and body composition, all modulated by maturity status; and (4) intervention pathways, where maturity-informed grouping, scaling adjustments, and bio-banding strategies are applied to promote equity, sustain participation, and enhance evaluation accuracy. By situating fitness assessment within this framework, the model emphasizes that maturity-sensitive approaches are essential for both valid measurement and effective intervention design in children and adolescents.

## 2. Literature Search Strategy

To ground this structured narrative review, we conducted a focused literature search to identify studies exploring how biological maturity can be integrated into physical activity programs and performance assessments for young people. Electronic databases, including PubMed, Scopus, and Web of Science databases, were searched using the following terms: ‘biological maturation’, ‘physical fitness’, ‘youth sport’, ‘peak height velocity’, ‘bio-banding’, ‘cardiorespiratory fitness’, ‘muscular strength’, and ‘body composition’. Searches were conducted in February 2026 and were not restricted by publication date, although priority was given to articles published after 2010. Reference lists of included articles were also screened to identify additional relevant sources. Articles were included if they were peer-reviewed, written in English or French, and addressed relationships between maturity indicators and fitness or physical activity outcomes in children and adolescents aged 8–19 years. The minimum quality thresholds applied were: peer-reviewed publication in an indexed journal, sample size ≥ 30 participants, and use of a validated maturity assessment method. No formal quality appraisal tool (e.g., GRADE, ROBINS) was applied, which is consistent with the narrative review design; this decision is briefly justified below.

A structured narrative review design was deliberately selected for three interrelated reasons. First, the overarching objective was integrative rather than estimative: we sought to synthesize evidence across multiple interconnected domains—including somatic growth, hormonal regulation, maturity assessment methodology, sex-specific fitness patterns, and applied intervention design. Given the substantial heterogeneity in maturity assessment methods, study populations, fitness outcomes, and analytical approaches across the available literature, pooling effect estimates through formal meta-analytic procedures would risk producing misleading summary statistics. Second, the present contribution aims to develop a unifying conceptual model situating biological maturity within a continuum from hormonal regulation through to public health intervention—a theoretical and synthesis-oriented objective better served by narrative synthesis. Third, several high-quality systematic reviews and meta-analyses have already addressed specific sub-questions within this domain [12,13,14], and these are explicitly incorporated into our synthesis. The added value of this review, therefore, lies in cross-domain integration and the development of a practitioner-oriented conceptual framework. The absence of formal quality appraisal is acknowledged as a limitation of the narrative design (see Section 7.4).

## 3. Somatic Growth During Adolescence

### 3.1. Individual Variability in Maturation Timing

One of the most visible characteristics of puberty is the adolescent growth spurt. In girls, the peak height velocity (PHV), the point at which the rate of stature increase is maximal, generally occurs between approximately 11.5 and 12.5 years of age. In boys, PHV typically occurs later, around 13.5 to 14.5 years of age. Average growth rates during this phase are approximately 6–7 cm per year in females and 8–10 cm per year in males, contributing to sex differences in adult height [15,16].

Growth during adolescence is not limited to stature. Body mass also increases substantially as both fat-free mass and fat mass accumulate. Boys generally experience greater gains in lean tissue, particularly skeletal muscle, whereas girls typically show relatively larger increases in fat mass during the pubertal transition. These compositional differences have direct implications for several aspects of physical fitness, including strength, power, and endurance performance [17,18].

Longitudinal investigations such as the Fels Longitudinal Study and the Saskatchewan Growth and Development Study have documented typical patterns of adolescent growth [19]. These studies highlight the considerable variability in growth timing among individuals and confirm that chronological age alone provides an incomplete representation of biological development during this period.

The mechanistic links between somatic growth and physical conditioning operate through several interconnected pathways [20]. Increases in stature and limb length augment mechanical leverage and stride length, thereby improving sprint speed and jump height through biomechanical advantage. Gains in lean body mass directly increase the physiological substrate for force production, underpinning improvements in muscular strength and power [21]. Changes in body composition—particularly the sex-divergent trajectories of lean versus fat mass accrual—modulate weight-bearing performance, locomotor economy, and relative aerobic capacity [22]. Understanding these pathways is essential for interpreting maturity-related variation in fitness test performance.

### 3.2. Skeletal Development and Bone Age

Skeletal age, also known as bone age, reflects the biological maturity of the skeletal system. It is traditionally assessed through radiographic examination of the hand and wrist using standardized reference atlases such as the Greulich Pyle or Tanner–Whitehouse methods. Skeletal age may deviate from chronological age by several years in either direction, illustrating the large variability in maturation among youth of the same age [23].

During puberty, substantial increases in bone mineral content and bone density occur. Peak bone mass accrual generally occurs shortly after PHV. The coordination between linear growth and skeletal mineralization is crucial because it influences long-term bone strength and fracture susceptibility [24].

Growth plates, located at the ends of long bones, remain open during much of adolescence. Before these plates close, they represent potential injury sites, particularly in physically active youth exposed to repetitive mechanical loading. Understanding skeletal maturity, therefore, plays an important role in managing training loads and preventing injuries in young athletes [25].

### 3.3. Hormonal Regulation of Growth

Adolescent growth is governed by complex hormonal interactions. The growth hormone (GH)–insulin-like growth factor-1 (IGF-1) axis is central to stimulating longitudinal bone growth and increasing lean body mass. Pubertal sex hormones further amplify these processes. Testosterone in males promotes substantial increases in muscle mass and strength, whereas estrogen in females contributes to skeletal maturation and changes in body composition [26].

Energy availability also influences the onset and progression of puberty. Hormones such as leptin, produced by adipose tissue, signal the hypothalamic-pituitary axis about adequate energy reserves and help initiate pubertal development. Consequently, nutritional status and body composition can affect both the timing of puberty and subsequent fitness development [27,28].

## 4. Biological Maturity Assessment Methods

### 4.1. Overview of Assessment Methods

Several approaches have been developed to evaluate maturity status in youth populations. The selection of an appropriate method depends on the research context, available resources, and the required level of precision. Various methods for estimating growth and maturity have been proposed. A review and recent studies have highlighted the need for more and better research in this field, due to the shortcomings of most approaches used [12,29,30]. Table 1 provides a comparative overview of the four principal methods currently used in research and applied settings.

#### 4.1.1. Skeletal Age

Skeletal age assessment remains the most accurate clinical indicator of biological maturation. By comparing radiographic images of the hand and wrist with standardized reference atlases, practitioners can estimate the stage of skeletal development. Despite its accuracy, this technique is not always feasible for large-scale research or field-based programs due to cost, equipment requirements, and ethical considerations related to radiation exposure [23].

#### 4.1.2. Peak Height Velocity and Maturity Offset

Age at PHV is a commonly used landmark for identifying maturity timing in longitudinal studies. Adolescents who experience PHV earlier than the population average are categorized as early matures, whereas those reaching PHV later are considered late matures. Prediction equations based on anthropometric variables, such as height, sitting height, leg length, and body mass, have been developed to estimate the number of years an individual is before or after PHV [35]. These non-invasive methods are practical for field settings but carry important methodological caveats. The typical prediction error of maturity offset equations is approximately ±1 year, with reduced accuracy at extreme maturity offsets [11,12]. Furthermore, the original equations were derived predominantly from White European and Canadian samples, limiting their generalizability to diverse ethnic and geographic populations. Practitioners should acknowledge these limitations when applying PHV-based classifications in research or applied settings.

#### 4.1.3. Percentage of Predicted Adult Stature

Another non-invasive approach involves estimating the proportion of predicted adult stature already attained by an adolescent. Using information about the child’s current height and weight, along with parental heights, prediction models can estimate eventual adult stature and thereby provide a maturity indicator. This method offers a continuous measure that is practical for school and sport environments [10,34].

#### 4.1.4. Comparison of Methods and Methodological Implications

A critical consideration for practitioners is that different maturity assessment methods do not always produce equivalent classifications. Monasterio et al. [30] demonstrated notable inter-method inconsistency in maturity classification when comparing skeletal age, Tanner staging, maturity offset, and percentage of predicted adult stature in elite male youth soccer players. PHV offset and Tanner staging, in particular, do not always yield equivalent maturity classifications, especially at transitional pubertal stages, and the degree of agreement varies by age range and sex. In high-stakes talent selection environments, the selected method can meaningfully influence which players are identified as early or late maturing, with direct consequences for developmental pathways and career outcomes. Flores et al. [31] subsequently developed skeletal-age prediction models specific to male soccer players, stratified by maturity status, reinforcing that methodological choice is not a neutral decision. Practitioners should therefore select assessment methods appropriate to their context, acknowledge inherent limitations, and interpret classifications with appropriate caution.

### 4.2. Patterns of Height and Weight Gain

Within any cohort of adolescents of the same chronological age, substantial differences in maturation timing exist. The typical range of variation around the average age at PHV spans roughly 2 years in either direction. Consequently, youth within a single school grade may represent almost the entire spectrum of pubertal development [16].

Maturational timing can influence psychosocial experiences and physical performance. Early-maturing boys often display advantages in strength, speed, and power during adolescence, whereas early-maturing girls may experience increased fat mass accumulation. Late-maturing adolescents may temporarily appear physically disadvantaged compared with peers, although many eventually reach levels of adult performance that are similar or even superior [36].

### 4.3. Emerging Roles of Artificial Intelligence and Machine Learning

Artificial intelligence (AI) and machine learning (ML) methods are increasingly relevant to biological maturity assessment and represent a priority area for future methodological development. The following evidence is presented to illustrate this potential and to contextualize the research directions proposed in Section 9. Deep convolutional neural networks trained on large radiographic datasets have demonstrated skeletal age estimation accuracy comparable to, and in some cases exceeding, that of expert radiologists [37,38]. These automated systems process hand and wrist radiographs within seconds, producing objective, reproducible maturity estimates while substantially reducing inter-rater variability [38].

The BoneXpert algorithm, one of the earliest and most validated automated systems, introduced the concept of unsupervised, software-driven bone age reading and has since been adopted in multiple clinical and research settings [37]. Beyond imaging, non-invasive ML pipelines using anthropometric inputs, wearable sensor streams, and GPS-derived movement data have been explored for field-based maturity classification [31,37,38]. When trained on sex-balanced, ethnically diverse datasets, such approaches have produced maturity offset predictions with errors approaching those of established equations, while offering practical scalability for school and sport academy settings [11,31,35].

The emergence of generative AI (GenAI) and large language models (LLMs) introduces further possibilities for individualized, maturity-aware exercise prescription and athlete development planning. Dergaa et al. [39,40] have documented both the capabilities and the substantive limitations of AI-assisted tools in sports medicine and exercise science, consistently emphasizing that AI outputs require rigorous human oversight. In the specific context of youth populations, particular caution is warranted: training data bias, limited generalisability across ethnic groups, and the opacity of complex model architectures each pose meaningful challenges. Algorithmic maturity assessments carry ethical implications when used in high-stakes decisions, such as team selection or talent deselection, and their use must be governed by transparent reporting standards, prospective validation across diverse cohorts, and clear protocols distinguishing AI-assisted from AI-determined conclusions. At present, these AI-based approaches should be considered complementary tools rather than replacements for established biological maturity assessment methods, pending broader external validation across diverse populations.

## 5. Physical Fitness in Adolescents

The five fitness components reviewed in this section span both health-related fitness (cardiorespiratory endurance, muscular strength/endurance, flexibility, body composition) and performance-related fitness (speed, agility, power). This inclusive framework reflects the dual scope of the review, which addresses both public health programming and sport development contexts. Assessment examples are drawn from established batteries, including EUROFIT, FitnessGram, and ALPHA-Fitness, and are identified by battery where applicable. Organized from most to least maturity-dependent, the five components are: (1) muscular strength and power (strongest and most consistent maturity effect, particularly in males); (2) speed and sprint performance; (3) cardiorespiratory fitness (maturity effect is scaling-method dependent); (4) body composition (a structural maturity outcome and performance mediator); and (5) flexibility (complex, non-linear relationship with maturation).

The ordering of fitness components reflects the relative magnitude and consistency of biological maturity effects reported across the literature, rather than direct quantitative comparisons of absolute effect sizes.

Table 2 provides a structured overview of the five-core fitness components examined in this review, summarizing assessment methods, sex- and developmental timing-related maturity effects, and key considerations for practitioners.

### 5.1. Cardiorespiratory Fitness

Cardiorespiratory fitness (CRF), frequently quantified as maximal oxygen uptake (VO_2_max), is a key indicator of cardiovascular health in youth. Higher CRF levels are associated with reduced cardio metabolic risk, improved psychological well-being, and better academic outcomes [7]. CRF is a core component of health-related fitness and is assessed in both EUROFIT (20 m shuttle run) and FitnessGram (PACER test) batteries.

Absolute VO_2_max typically increases during adolescence as cardiac output, hemoglobin concentration, and muscle mass rise. In boys, these increases accelerate during puberty due to testosterone-related physiological adaptations. In girls, improvements occur more gradually, and relative VO_2_max may decline slightly as body fat increases [47].

When examining CRF in relation to maturity status, early-maturing boys often demonstrate higher absolute VO_2_max values compared with late-maturing peers of the same age. However, when oxygen uptake is expressed relative to body mass, these differences often diminish, highlighting the importance of appropriate scaling methods when interpreting fitness data [7].

Field-based CRF tests, such as the 20 m shuttle run or 6-min run, are widely used in school and sport contexts due to their practicality. Research using these assessments consistently demonstrates that biological maturity contributes significantly to performance differences among adolescent boys.

These findings collectively underscore the importance of incorporating maturity status as a covariate when interpreting CRF norms or comparing performance between individuals of the same chronological age, as failure to do so risks systematically underestimating the fitness of late-maturing youth.

### 5.2. Muscular Strength and Power

Muscular strength increases markedly during adolescence, particularly in males. The surge in testosterone during mid-puberty stimulates muscle hypertrophy and neuromuscular adaptations, leading to rapid improvements in maximal strength. Peak rates of strength development typically occur approximately one to one-and-a-half years after PHV [41].

In females, strength gains are generally smaller in magnitude and primarily reflect increases in lean mass and neuromotor coordination rather than large increases in muscle size. As a result, sex differences in absolute strength become increasingly pronounced during adolescence [8,48]. Normative standards derived predominantly from male samples should not be directly applied to female populations when interpreting strength test results in youth sport or physical education settings.

Commonly used strength assessments in youth research include handgrip dynamometry, jump tests, and isokinetic strength measurements. Numerous studies show that maturity status strongly predicts performance on these tests, particularly among boys [49,50,51]. Muscular power, which combines force and movement velocity, follows similar developmental patterns, with substantial increases in peak power output during male puberty and more gradual improvements in females [16].

### 5.3. Flexibility

Flexibility typically follows a different developmental trajectory compared with strength or endurance. Periods of rapid skeletal growth may temporarily reduce flexibility because bones lengthen more quickly than surrounding muscles and connective tissues can adapt. This imbalance can increase susceptibility to musculoskeletal injury during the growth spurt.

Field assessments such as the sit-and-reach test are commonly used to evaluate flexibility in adolescents. In general, girls demonstrate greater flexibility than boys throughout adolescence, partly due to differences in body composition and hormonal influences on connective tissue [44].

### 5.4. Speed, Agility, and Motor Performance

Running speed and agility improve considerably during adolescence as limb length, neuromuscular coordination, and muscular power increase. In boys, improvements in sprint performance are closely associated with gains in lean mass during puberty.

Agility, defined as the ability to change direction rapidly and efficiently, depends on multiple physical attributes, including strength, coordination, and body dimensions. Although early-maturing boys often demonstrate advantages in agility tasks, the relationship between maturity and agility is less straightforward than that observed for pure strength measures [42,52].

Rapid growth during puberty can also temporarily disrupt motor coordination due to changes in body proportions. This phenomenon, sometimes referred to as adolescent awkwardness, highlights the importance of continued skill development during this stage of life [43].

### 5.5. Body Composition

Body composition is one of the most maturation-sensitive domains of physical fitness in adolescents [53]. Within the health-related fitness framework, body composition is classified as a structural indicator of physiological readiness and health status, rather than a performance test per se. Its inclusion alongside functional fitness components in this review is justified by its dual role: as a direct outcome of maturation-driven hormonal changes, and as a mediating variable through which maturation exerts its primary influence on functional performance. As biological maturity progresses, changes in fat mass, lean mass, and bone mineral density alter not only how the body looks but also how it moves, responds to training, and compares across individuals of the same chronological age [2,11].

#### 5.5.1. Hormonal Drivers of Maturation-Linked Body Composition Change

The compositional changes of puberty are orchestrated by a cascade of hormonal events that unfold in a sex-specific manner [26]. In boys, the pubertal surge in testosterone—mediated through the hypothalamic-pituitary-gonadal axis—drives substantial increases in skeletal muscle mass and lean body mass. This anabolic environment promotes satellite cell proliferation, myofibrillar protein synthesis, and progressive increases in muscle cross-sectional area, all of which accelerate markedly in the 12 to 18 months surrounding peak height velocity (PHV) [16,41]. Simultaneously, relative fat mass typically stabilizes or declines as lean tissue accumulation outpaces adipose deposition.

In girls, estrogen and progesterone govern a fundamentally different compositional trajectory. Estrogen promotes adipogenesis in the gluteofemoral and subcutaneous depots, resulting in characteristic increases in total and regional fat mass during and after PHV [18,26]. Although lean mass also increases in girls, these gains are substantially smaller in magnitude and are driven more by neuromotor maturation and limb lengthening than by myofibrillar hypertrophy. The net result is a progressive divergence in fat-free mass and fat mass between the sexes that begins at mid-puberty and widens throughout late adolescence [17,53].

Beyond the primary sex hormones, the growth hormone (GH)–insulin-like growth factor-1 (IGF-1) axis plays an integrative role. GH and IGF-1 stimulate both longitudinal bone growth and lean mass accrual in both sexes, with peak secretion coinciding with the adolescent growth spurt [26]. Changes in leptin—a hormone produced by adipose tissue in proportion to fat mass—also interact with the reproductive axis, signaling energy availability and potentially amplifying the fat-mass increases observed in early-maturing girls [27,28]. This hormonal cross-talk means that maturation, nutrition, and body composition are deeply intertwined, and that any assessment of body composition in adolescents must be interpreted in light of the maturational stage reached.

#### 5.5.2. Maturation Timing and Sex-Specific Compositional Trajectories

Within any cohort of same-aged adolescents, maturation timing introduces considerable compositional heterogeneity that chronological-age norms cannot adequately capture [3]. In boys, early maturation is associated with an earlier and more pronounced lean mass advantage. Early-maturing boys enter the testosterone-driven hypertrophic phase earlier, accumulate greater muscle mass relative to late-maturing peers of the same chronological age, and consequently demonstrate superior strength and power performance throughout mid-adolescence [9,54,55]. This advantage is largely transient: late-maturing boys typically converge toward a similar adult body composition once their own pubertal hormone surge occurs, although some long-term differences in muscle fiber characteristics and lean mass distribution may persist [36].

Among girls, the compositional consequences of early maturation are more complex and, in many performance contexts, less advantageous. Early-maturing girls accumulate fat mass more rapidly and at a younger chronological age than their average- or late-maturing peers [13]. This accelerated adiposity has several interconnected consequences for fitness. First, weight-bearing performance—including running economy, vertical jump height, and agility—is directly impaired by excess non-functional mass [56]. Second, the increase in total body mass without a commensurate rise in aerobic capacity causes relative VO_2_max (expressed per kilogram of body mass) to decline, even when absolute cardiorespiratory fitness is adequate or above average [7,47]. Third, repeated performance comparisons that do not account for these maturity-driven compositional differences risk yielding inaccurate and potentially demoralizing fitness classifications for early-maturing girls [57].

Late-maturing girls, by contrast, retain a lighter and leaner body composition for longer, which may confer temporary advantages in endurance and weight-bearing activities. However, they may also exhibit lower absolute muscular strength and reduced bone mineral density relative to early-maturing peers during the same developmental window, illustrating that the compositional effects of maturation are multidirectional and context-dependent [13,53].

Understanding sex-specific differences in maturation timing is particularly important. Girls experience PHV approximately 1.5–2 years earlier than boys (approximately 11.5–12.5 vs. 13.5–14.5 years), meaning that the estrogen-driven adipogenesis and compositional changes described above occur at younger chronological ages in females [58]. The divergence in body composition between early- and late-maturing girls is most pronounced approximately 2 years after female PHV. These earlier and compositionally distinct changes mean that fitness evaluations applied to mixed-maturity female groups are particularly susceptible to misclassification, and that maturity adjustment is at least as important—arguably more so—for female populations than for males.

#### 5.5.3. Body Composition Around Peak Height Velocity

The period immediately surrounding PHV represents a particularly critical window for body composition assessment and monitoring [16]. During this phase, skeletal growth accelerates sharply, creating a temporary imbalance in the relative rates of bone elongation, muscle growth, and connective tissue adaptation. In boys, the rapid increase in limb length during the PHV phase precedes the major gains in muscle mass and strength, which typically peak approximately one to one-and-a-half years after PHV [41]. This asynchrony between skeletal and muscular development creates a transitional period of reduced relative strength and altered body proportions, which can transiently impair performance and increase injury risk [14,25].

In girls, the PHV phase is accompanied by a hormonally driven acceleration in fat mass deposition that occurs earlier than in boys. Longitudinal data indicate that the divergence in body composition between early- and late-maturing girls is most pronounced approximately 2 years after female PHV, after which late-maturing girls begin their own pubertal fat mass accrual [53]. Practitioners assessing body composition in this age group should therefore record and report maturity status ideally as years before or after PHV using validated prediction equations [11,33] or as a percentage of predicted adult stature [10], alongside all body composition metrics to appropriately contextualize observed values.

#### 5.5.4. Body Composition and Physical Performance: Maturation-Mediated Pathways

The influence of body composition on physical performance during adolescence cannot be disentangled from maturation, because maturation is the primary driver of the compositional environment in which performance occurs [9,13]. In boys, the testosterone-mediated lean mass gains of puberty create direct mechanistic pathways to superior muscular strength, sprint speed, and jump performance that are largely unavailable to pre-pubertal or late-maturing individuals of the same chronological age [8,16,55]. These maturation-mediated compositional advantages explain a substantial proportion of the within-cohort performance variance observed in youth sport and physical education settings.

In girls, the relationship is more nuanced. The fat mass accrual associated with early maturation is physiologically normative but functionally constraining in performance contexts that require moving body weight against gravity or over distance [13,47]. Early-maturing girls may therefore present a paradox in fitness testing: their absolute muscular strength may be superior to that of late-maturing peers—because lean mass has also increased alongside fat mass—while their relative endurance and agility scores appear lower. Failing to account for maturation when interpreting these data risks misclassifying physically healthy, normal-maturing girls as “unfit” based on normative standards that do not reflect their developmental context [7,57].

The implications for talent identification are equally significant. Body composition-related performance differences between maturity groups are largest at mid-puberty, when early matures have progressed well through their compositional transformation, while late matures remain in a pre- or early-pubertal state [9,59]. In competitive youth sport, these transient compositional advantages can strongly influence coach perceptions and selection decisions, contributing to the systematic over-representation of early-maturing individuals in talent pathways and the premature exclusion of late-maturing athletes with equal or greater long-term potential [59,60].

#### 5.5.5. Body Composition Assessment: Methods and Maturation-Sensitive Applications

Body composition can be assessed using a range of methods, each presenting distinct trade-offs between accuracy, cost, feasibility, and sensitivity to maturation-related change [45,46]. The choice of method is not maturation-neutral: some tools are better suited to detecting the specific compositional shifts driven by puberty, while others are inadequate for capturing the dynamic changes occurring during rapid growth phases.

Dual-energy X-ray absorptiometry (DXA) is considered the gold standard for quantifying fat mass, lean mass, and bone mineral density in research settings [45]. Its ability to simultaneously assess all three compartments makes it particularly valuable for longitudinal maturity-tracking studies, where the coordination among lean mass accrual, changes in fat mass, and skeletal mineralization around PHV is of central interest. However, cost, radiation exposure, and equipment requirements limit its application to clinical and laboratory settings.

Bioelectrical impedance analysis (BIA) provides a more practical alternative for field-based monitoring and is increasingly used in school and academy contexts [45,46]. While less precise than DXA—particularly during rapid hydration shifts associated with pubertal growth—BIA can serve as a cost-effective tool for serial monitoring when population-specific, maturity-stratified prediction equations are applied. It is important that practitioners recognize that standard adult equations may not accurately reflect the specific tissue composition of pubescent individuals, particularly during the accelerated growth phases surrounding PHV.

Skinfold anthropometry remains widely used for its accessibility, low cost, and minimal equipment requirements. In maturation research, skinfold measurements can capture the subcutaneous fat accumulation characteristic of early maturation in girls and provide information about regional fat distribution that BMI and impedance measures cannot [46]. Precision depends heavily on assessor skill and site selection, and measurement error becomes particularly important during rapid growth when tissue properties change quickly.

Body mass index (BMI) is routinely collected in population health surveillance, but it is an inadequate tool for maturity-sensitive body composition assessment [53]. Because BMI cannot distinguish between fat and lean mass, it systematically misrepresents the compositional profile of adolescents at different maturational stages: an early-maturing boy with high lean mass and an early-maturing girl with elevated fat mass may present identical BMI values despite radically different body composition profiles with opposing implications for fitness and health. Practitioners should exercise caution when using BMI as a proxy for fatness in pubertal populations and should supplement it with at least one valid body composition measure.

Waist circumference and the waist-to-height ratio offer simple, maturation-sensitive indices of central adiposity that are directly relevant to metabolic health monitoring in adolescents [27,28]. Central fat accumulation, which can accelerate in early-maturing girls following PHV, is more strongly associated with cardiometabolic risk than total fat mass and may not be adequately captured by whole-body composition methods. These measures, therefore, complement DXA or BIA in comprehensive, health-oriented assessments.

## 6. Maturity-Related Variation in Physical Fitness

### 6.1. Sex-Specific Patterns

Biological maturation affects fitness differently in males and females. Table 3 compares maturity-related fitness patterns by sex across the five fitness components reviewed, illustrating distinct developmental trajectories and their practical implications.

The influence of biological maturation on fitness differs substantially between males and females. In boys, maturation exerts a strong positive influence on most physical performance variables during adolescence. Early-maturing boys often outperform later-maturing peers in strength, power, and sprinting tasks [9,54,55].

Among girls, the relationship between maturation and fitness is more complex. Earlier maturation is frequently associated with greater fat mass, which may hinder performance in weight-bearing activities such as running or jumping. As a result, early-maturing girls do not always show the same performance advantages as boys [13].

The relative paucity of longitudinal studies tracking maturation-fitness relationships in female adolescents represents a notable gap in the literature. Most existing data derive from cross-sectional designs in which early-maturing girls demonstrate lower cardiorespiratory fitness scores relative to body mass, yet similar or superior absolute muscular strength compared with late-maturing age peers [13]. Fitness evaluation tools and normative standards developed primarily from male samples may not translate directly to female populations. Future research should prioritize sex-stratified longitudinal designs that capture the full pubertal trajectory in girls, including interactions between hormonal changes, fat mass accrual, and fitness development.

Sport-specific evidence from handball and basketball further illustrates these sex-differentiated patterns in competitive youth populations. Aouichaoui et al. [54] demonstrated that biological maturity status significantly predicted physical performance across strength, speed, and endurance domains in male handball players aged 13 to 19 years, even after controlling for chronological age, reinforcing the necessity of maturity-adjusted normative standards in youth sport evaluation. Similarly, Gryko [55] reported substantial maturity-related performance disparities among male Polish youth basketball players aged 13 to 15 years, with early maturation demonstrating systematic advantages across multiple performance indices that could not be attributed solely to chronological age. These findings underscore that the practical consequences of ignoring maturity status in fitness evaluation extend beyond physical education into organized competitive sport, where selection and development decisions carry long-term implications for athlete retention and career progression.

### 6.2. Relative Age Effect

The relative age effect (RAE) refers to the consistent overrepresentation of athletes born early in the selection year within competitive youth sport. This pattern is partly attributable to biological maturation: within a single-age cohort, chronologically older athletes are more likely to be biologically advanced, gaining temporary advantages in strength, stature, and power that can shape coaches’ perceptions and selection outcomes [61]. Soccer provides the most robust evidence for the RAE among team sports, with early-born players consistently overrepresented from grassroots to elite academies across diverse national settings. Importantly, the RAE in soccer extends beyond birth date alone; it interacts with biological maturation so that early-maturing players—irrespective of birth quartile—are preferentially selected and retained in high-performance pathways, while late-maturing players with comparable or superior technical skills are often excluded [59].

Bio banding—grouping athletes by estimated biological maturity rather than chronological age—offers a practical, theory-informed approach to mitigate these structural biases and promote more equitable developmental environments [60]. In bio-banded settings, late-maturing athletes compete against physically similar peers, allowing coaches and scouts to evaluate technical and tactical skills without the confounding influence of maturational disparities. Beyond competition, bio-banding supports talent identification, load management, and strength and conditioning programming, where maturity-matched training stimuli help reduce overuse injury risk and optimize the timing of physical development interventions. Evidence from soccer illustrates the potential for broader application of bio banding across youth team sports, physical education, and any performance context where chronological age grouping may disadvantage later-maturing individuals.

### 6.3. Tracking of Fitness into Adulthood

Research indicates that certain aspects of physical fitness track moderately from adolescence into adulthood. However, the degree of tracking is influenced by behavioral factors such as physical activity participation as well as biological factors, including maturation timing. Late-maturing adolescents who appear disadvantaged in early adolescence often show substantial improvement once their growth spurt occurs [62].

## 7. Methodological Considerations

### 7.1. Challenges in Assessing Maturity

Evaluating biological maturity in research settings presents several methodological challenges. While skeletal age provides the most accurate assessment, its use is often limited in large studies. Non-invasive prediction methods are more practical but introduce estimation errors [31].

Additionally, many existing studies employ cross-sectional designs that cannot fully capture the dynamic nature of growth and maturation. Longitudinal investigations are better suited for understanding developmental trajectories but require significant resources and long-term participant follow-up [63].

### 7.2. Normative Reference Data

Fitness reference standards for youth are typically organized according to chronological age. However, because biological maturity significantly influences physical performance, age-based norms alone may misrepresent an individual’s true fitness status. Incorporating maturity-adjusted standards could improve the accuracy of performance evaluations [64].

Currently available normative databases, such as EUROFIT, FitnessGram, and the ALPHA-Fitness battery, are organized primarily by chronological age and sex, without integration of maturity status [65]. An intermediate approach to maturity adjustment, already implemented in some national physical education systems, involves stratifying normative references by birth quartile within a calendar year. This approach partially mitigates the confounding effects of chronological age variation within cohorts and represents a practically accessible step toward more maturity-sensitive assessment. However, birth-quartile stratification does not directly account for biological maturity within quartiles, and its effectiveness is therefore limited compared with approaches using validated maturity indicators. Efforts to develop fully maturity-stratified normative databases remain a high priority for applied exercise physiology and public health research [66,67].

### 7.3. Physical Activity as a Confounding Factor

Habitual physical activity strongly influences physical fitness in adolescents. Early-maturing individuals may be more likely to participate in organized sport due to their physical advantages, which can further enhance their fitness levels. Conversely, late-maturing adolescents may withdraw from physical activity if repeated comparisons with more mature peers are discouraging [68].

### 7.4. Limitations of the Narrative Review Approach

This narrative review carries inherent methodological limitations. Unlike systematic reviews, narrative syntheses are vulnerable to selection bias, as study inclusion and emphasis may reflect authors’ familiarity with the particular literature streams. The absence of formal quality appraisal or risk-of-bias assessment means methodologically weaker studies may have been weighted equally with more rigorous work. Furthermore, quantitative synthesis of effect sizes was beyond this review’s scope, limiting the ability to draw definitive conclusions about the magnitude of maturation effects on specific fitness components.

An additional implementation limitation, relevant to the school-based applications discussed in Section 8.1, is that maturity-adjusted norms require appropriately trained personnel for valid application. PE teachers are not typically qualified to estimate biological maturity directly. Without structured professional development and clear institutional guidelines, maturity-adjusted norms could inadvertently facilitate pupil classification or grading—contrary to the intent of health-related fitness testing in educational contexts, where assessment is intended to inform individual progress rather than rank pupils. Any implementation of maturity-sensitive assessment in schools must be accompanied by teacher training, clear communication of purpose, and explicit policies prohibiting the use of such assessments for grade-based classification.

To address these constraints, we implemented a structured search across three major databases, applied pre-specified inclusion criteria, and anchored our synthesis in findings from existing systematic reviews and meta-analyses where possible. Future research should prioritize systematic reviews with meta-analysis focused on specific maturation–fitness relationships, particularly among female adolescents and across diverse ethnic and socioeconomic contexts.

## 8. Public Health and Applied Implications

Table 4 summarizes practical implications for applying maturity-informed strategies across the five primary settings reviewed.

### 8.1. School-Based Physical Education

Fitness testing is widely implemented in school physical education (PE) programs. However, comparing students solely by chronological age may lead to misleading interpretations of performance [57].

Incorporating maturity-sensitive evaluation strategies could create more supportive and motivating learning environments. Specific practical strategies for school-based implementation include: (1) adoption of maturity-stratified norms within existing batteries such as FitnessGram or EUROFIT; (2) training PE teachers to use validated non-invasive maturity estimation tools (maturity offset equations or % predicted adult stature); (3) shifting assessment philosophy from normative ranking to criterion-referenced and individualized progress tracking; and (4) use of qualitative observation during PHV phases to detect adolescent awkwardness and modify skill and performance expectations accordingly. It is essential that these strategies are accompanied by structured teacher professional development programs and clear institutional policies, given the risk identified in Section 7.4 that maturity-adjusted norms could be misapplied in under-resourced or poorly supported school contexts.

### 8.2. Talent Identification in Youth Sport

Talent identification programs frequently rely on physical performance indicators strongly influenced by biological maturity. Without appropriate adjustment, these systems tend to favor early-maturing athletes and may overlook individuals with substantial long-term potential. Strategies such as bio-banding and maturity-adjusted evaluation frameworks have been proposed to mitigate this bias [59].

Soccer offers the most methodologically robust evidence base for maturity-informed talent identification. Common physical screening measures—sprint speed, jump height, aerobic capacity—are strongly confounded by maturity status, and without adjustment, selection processes systematically favor early-maturing players [59]. Several sport federations and national systems have moved beyond conceptual proposals and are now implementing bio-banding in practice. The English Football Association piloted bio-banded tournaments from 2016 onward, reporting that late-maturing players demonstrated greater technical skill expression and improved psychosocial outcomes when competing against peers of similar physical maturity. The Welsh Rugby Union has implemented bio-banding in age-group squads, reporting comparable benefits. UEFA Youth League formats have been explored for maturity-adjusted competition structures, and Australian talent programs have incorporated maturity-informed selection criteria into national pathways. In each context, reported benefits include reduced relative age bias, greater visibility of technical and tactical qualities in late-maturing players, and improved motivation. Logistical challenges—including resistance from coaches accustomed to chronological-age structures and the resource requirements of maturity monitoring—are consistently noted and should be anticipated in implementation planning. These examples serve as transferable models for other sports and national systems seeking to reduce maturity bias in talent identification.

### 8.3. Injury Prevention

Periods surrounding the pubertal growth spurt represent times of increased injury vulnerability. Rapid skeletal growth can temporarily reduce flexibility and alter movement mechanics. Monitoring maturation status may help coaches and practitioners adjust training loads and prevent overuse injuries [14,69].

### 8.4. Addressing Physical Inactivity and Obesity

Rising levels of physical inactivity and obesity among adolescents represent major public health concerns. Interventions designed to promote physical activity should recognize the diversity of biological development among youth. Programs that emphasize personal improvement rather than peer comparison may help maintain motivation across different maturity levels [14,57,68,70].

## 9. Future Research Directions

Future research should prioritize large longitudinal studies examining the interactions among growth, maturation, physical activity, and multiple fitness components from early adolescence into adulthood. Improved non-invasive maturity assessment tools are also needed to enhance field-based research and applied practice.

Specifically, several key research priorities are proposed: (1) Development and validation of sex-specific, maturity-adjusted normative standards for the complete battery of health-related fitness tests. This is the most immediate need, as the absence of such standards represents the primary obstacle to equitable fitness assessment in school and sport contexts. Large multinational longitudinal datasets are required. (2) Longitudinal investigations tracking fitness trajectories from early puberty through late adolescence, particularly in female populations. Given the documented gap in evidence on female-specific maturation–fitness relationships, this represents a critical priority, with particular focus on interactions among hormonal changes, fat mass accrual, and functional fitness development across the full pubertal trajectory. (3) Rigorous intervention trials evaluating the effectiveness of bio-banding and maturity-informed curricular designs on physical activity levels, fitness outcomes, and psychological well-being in school and sport contexts. (4) The integration of wearable sensor technologies and digital health platforms to enable continuous, objective monitoring of physical activity patterns and maturation-related physiological changes in real-world settings. (5) Examination of the interaction between nutritional status, sleep quality, and maturation timing on physical fitness development, given the increasing prevalence of nutritional deficiencies and sleep disruption in adolescent populations. (6) AI and ML applications in maturity research deserve structured investment as a priority area. Specifically: development and external validation of deep-learning models for automated bone age estimation across ethnically diverse and globally representative pediatric samples; non-invasive ML classifiers combining wearable biometrics with anthropometric predictors for large-scale screening; and—critically—responsible deployment frameworks for GenAI tools in exercise prescription and athlete development contexts, with human oversight, auditability, and algorithmic bias safeguards [39,40]. Standardized reporting guidelines for AI-assisted maturity assessment studies are currently absent, representing a key methodological gap that the field must urgently address.

## 10. Conclusions

Biological maturation profoundly influences the development of physical fitness during adolescence. Evidence is most consistent and strongest for muscular strength and power—particularly in males—where the testosterone-driven hormonal environment of puberty creates direct mechanistic pathways to performance advantages that are largely unavailable to pre-pubertal or late-maturing peers. Speed and sprint performance follow closely. For cardiorespiratory fitness, the maturity effect depends on the scaling method used: absolute differences diminish substantially when VO_2_max is expressed relative to body mass. For females, the relationship between maturation and fitness is more nuanced, with earlier maturation frequently associated with increased adiposity that attenuates weight-bearing performance and relative aerobic capacity. This pattern is physiologically normative but functionally constraining. Flexibility is the least consistently maturity-dependent component, with the direction of effect dependent on the phase of growth. Interpreting physical fitness outcomes without considering maturity status therefore risks inaccurate, potentially demoralizing conclusions, particularly for late-maturing youth and early-maturing girls.

Three evidence-based implementation strategies are recommended. First, the maturity offset, or percentage of predicted adult stature, should be adopted as a standard covariate in all youth fitness assessments, reported alongside chronological age in research and applied settings. Second, sex-specific, maturity-stratified normative databases should be developed for established fitness batteries (EUROFIT, FitnessGram, ALPHA-Fitness), drawing on multinational longitudinal data. Third, bio-banding should be implemented in youth sport development and talent identification programs, informed by the growing evidence base from soccer, rugby, and other team sports.

Adopting maturity-informed evaluation frameworks across research, education, and sport contexts would improve the fairness and accuracy of performance assessment. It may support more inclusive physical activity environments that encourage sustained participation among all adolescents, regardless of developmental timing. From a policy perspective, failing to account for biological maturity represents not merely a methodological limitation but a structural source of inequity in youth physical education and sport development systems. The conclusions of this review should be interpreted in light of the inherent limitations of the narrative design, specifically, the absence of formal quality appraisal and meta-analytic effect-size synthesis, which constrain the precision of estimates of the magnitude of maturation effects on specific fitness components. These limitations reinforce the need for the systematic reviews and longitudinal investigations identified as priority directions in Section 9.

## Figures and Tables

**Table 1:** Comparison of commonly used biological maturity assessment methods in youth populations.

Method	Maturity Indicator	Invasiveness	Setting Suitability	Key Limitation
Skeletal age (Greulich–Pyle; Tanner–Whitehouse) [23,31]	Bone age (years)	Invasive (X-ray)	Clinical only	Radiation exposure; high cost; requires a trained radiologist
Tanner staging (secondary sexual characteristics) [12,32]	Pubertal stage (I–V)	Semi-invasive (clinical exam or self-report)	Clinical/research	Categorical scale; potential self-report bias; examiner variability
Age at Peak Height Velocity (maturity offset equations) [11,12,30,33]	Years before/after PHV	Non-invasive (anthropometry)	Field/school/sport	Prediction error ±1 year; equations may not generalize across populations Inter-method inconsistency with Tanner staging, particularly at transitional pubertal stages
% Predicted Adult Stature (PAH method) [10,29,34]	Proportion of adult height attained (%)	Non-invasive (anthropometry + parental heights)	Field/school/sport	Requires accurate parental height data; assumes typical growth patterns

Notes: PHV, peak height velocity; PAH, predicted adult stature.

**Table 2:** Summary of physical fitness components, assessment methods, and maturity-related effects in adolescents. Ranked from most to least maturity-dependent.

Fitness Component	Common Assessment	Maturity Effect (Males/Females)	Developmental Timing	Key Considerations
Cardiorespiratory Fitness (VO_2_max)	20 m shuttle run; 6 min run; treadmill test	Absolute VO_2_max is higher in early-maturing boys; no consistent advantage in girls	Relative VO_2_max differences diminish when scaled to body mass	Scaling method critical; maturity status should be covariate in norm interpretation Part of EUROFIT and FitnessGram health-related fitness batteries [7]
Muscular Strength and Power (Most Maturity-dependent)	Handgrip; isokinetic tests; vertical jump	Strong positive effect in boys; smaller, less consistent effect in girls	Peak strength development ~1–1.5 years post-PHV in males	Testosterone-driven hypertrophy is the key mechanism in boys; neuromotor gains are primarily in girls [8,41]
Speed and Sprint Performance	10 m/30 m sprint; agility T-test; illuminous gates	Early-maturing boys demonstrate faster sprint times; it is less clear in girls	Limb length and lean mass gains drive improvements during puberty	Agility relationship is less straightforward than pure speed; adolescent awkwardness is possible [42,43]
Flexibility (Least Consistently Maturity-dependent)	Sit-and-reach test; goniometry	Temporary decline possible during rapid growth spurt	Girls are generally more flexible than boys across all maturity stages	Bone lengthening outpaces connective tissue adaptation; increased injury risk [44] during PHV
Body Composition	DXA; BIA; skinfold; BMI	Boys: lean mass increase; Girls: fat mass increase during maturation	Fat mass increase in early-maturing girls may impair weight-bearing performance	Method choice affects accuracy; DXA is the gold standard, but costly; BIA is practical for the field [45,46]

VO_2_max, maximal oxygen uptake; PHV, peak height velocity; DXA, dual-energy X-ray absorptiometry; BIA, bioelectrical impedance analysis; BMI, body mass index.

**Table 3:** Sex differences in maturity–fitness associations across physical fitness components in adolescents.

Fitness Component	Males	Females	Developmental Pattern	Applied Implication
Cardiorespiratory Fitness	Absolute VO_2_max advantage in early maturing; the effect diminishes when expressed per kg BM	Early maturation is associated with higher fat mass, which attenuates relative VO_2_max PHV occurs 1.5–2 years earlier in girls, making compositional effects appear at younger chronological ages	Relative (per kg) VO_2_max is the appropriate scaling metric for cross-maturity comparisons	Avoid absolute VO_2_max comparisons without maturity adjustment [7,47]
Muscular Strength	Large advantage; testosterone drives hypertrophy post-PHV; peaks ~1–1.5 year after PHV	Smaller gains; primarily neuromotor rather than hypertrophic; less maturity-dependent	Sex divergence in absolute strength widens markedly from mid-puberty onward	Female normative data insufficient; male-derived standards should not be applied to girls [8,48]
Speed and Power	Clear advantage in sprint and jump performance for early maturation	Less consistent; body fat increase may offset lean mass gains in weight-bearing tests	The performance gap between early/late maturation is greatest at mid-puberty	Bio-banding is most impactful during the peak divergence window [9,60]
Flexibility	Temporary reduction during PHV; recovery post-spurt	Superior throughout adolescence; less affected by growth spurt	Sex difference in flexibility widens from early to mid-puberty	Injury risk is elevated in both sexes during rapid growth, greater in boys [14,44]
Body Composition	Lean mass increases; fat mass is relatively stable or decreasing	Fat mass increases (especially early maturing); lean mass gains are smaller driven by estrogen-mediated adipogenesis in gluteofemoral and subcutaneous depots	Divergence begins approximately 2 years after the female PHV The earliest and most pronounced effects in early-maturing girls	Early-maturing girls may benefit from body composition-independent fitness norms [13,53]

PHV, peak height velocity; VO_2_max, maximal oxygen uptake; BM, body mass.

**Table 4:** Practical applications of maturity-informed strategies across key youth sport settings.

Setting	Key Challenge	Recommended Strategy	Specific Actions
School-Based Physical Education	Misclassification of fitness levels, demotivation of late-maturing students, and inappropriate peer comparison	Maturity-adjusted norms; emphasis on personal improvement; avoidance of rank-order comparisons	Adopt maturity-stratified versions of EUROFIT/FitnessGram; train PE teachers in maturity-sensitive feedback; shift assessment focus from ranking to individual progress [57] Implementation must be accompanied by professional development and institutional policies prohibiting grade-based fitness classification (see Section 7.4)
Youth Sport and Talent Identification	Systematic over-selection of early matures; premature exclusion of late-maturing talent; relative age effect	Bio-banding; maturity-adjusted performance benchmarks; longitudinal tracking	Implement bio-banding tournaments; adjust scouting metrics for maturity offset; combine physical and technical/tactical evaluation to reduce maturity bias [59,60] Examples of established bio-banding programs: English FA (from 2016), Welsh Rugby Union, UEFA Youth League explorations, and Australian talent programs—see Section 8.2 for outcomes
Injury Prevention and Load Management	Elevated injury risk during rapid growth; open growth plates are vulnerable to overuse; altered movement mechanics	Maturity-informed training load adjustment; flexibility and strength monitoring around PHV	Monitor maturity status longitudinally in academy athletes; reduce high-impact loading at peak PHV; implement targeted flexibility protocols during growth spurt [14,69]
Public Health and Obesity Prevention	Physical inactivity driven by repeated negative comparisons; higher dropout in late maturation; obesity prevalence rising	Maturity-inclusive programming; focus on intrinsic motivation; personalized goals	Design interventions that emphasize participation over performance; communicate individual progress trajectories; integrate maturity education into health promotion curricula [68,70]
Clinical and Research Settings	Age-based norms misrepresent the true fitness of individuals with advanced or delayed maturation	Maturity offset or PAH as covariates; sex-stratified reference data	Report maturity status alongside chronological age in all youth fitness studies; develop multinational maturity-stratified normative databases; standardize maturity assessment protocols [10,12,64]

PE, physical education; PHV, peak height velocity; PAH, predicted adult stature.

## Data Availability

No new data were created or analyzed in this study.
